# OMICS Approaches to Assess Dinoflagellate Responses to Chemical Stressors

**DOI:** 10.3390/biology12091234

**Published:** 2023-09-13

**Authors:** Alice Roussel, Vincent Mériot, Thierry Jauffrais, Véronique Berteaux-Lecellier, Nicolas Lebouvier

**Affiliations:** 1 ISEA, EA7484, Campus de Nouville, Université de la Nouvelle Calédonie, Noumea 98851, New Caledonia; alice.roussel17@gmail.com (A.R.); vincent.meriot@etudiant.unc.nc (V.M.); 2 Ifremer, IRD, CNRS, Univ. de la Réunion, Univ. de la Nouvelle Calédonie, UMR 9220 ENTROPIE, 101 Promenade Roger Laroque, Noumea 98897, New Caledonia; thierry.jauffrais@ifremer.fr; 3 CNRS, Ifremer, IRD, Univ. de la Réunion, Univ. de la Nouvelle Calédonie, UMR 9220 ENTROPIE, 101 Promenade Roger Laroque, Noumea 98897, New Caledonia; veronique.berteaux-lecellier@cnrs.fr

**Keywords:** dinoflagellates, microalgae, phytoplankton, protist, OMICs, transcriptomic, proteomic, metabolomic, integrated OMICs, chemical stressors, nutrient starvation, anthropogenic contamination, genome complexity

## Abstract

**Simple Summary:**

Dinoflagellates are important primary producers known to biosynthesize metabolites of interest and toxins and form Harmful Algae Blooms (HABs). Water conditions such as nutrient availability, anthropogenic contaminants or pH impact dinoflagellate toxin productions, and HABs’ formation remains unclear. In this review, we present the recent contributions of OMICs approaches to the investigation of dinoflagellate responses to water chemical stressors. Transcriptomic and proteomic studies highlight whole-cell strategies to cope with nutrient deficiencies. Metabolomic studies offer a great view of toxin, lipid and sugar productions under stressors. However, the confrontation of different OMICs studies is tedious, as approaches are conducted in different species. As for other model organisms, it would be interesting to use multi-OMIC approaches to build a complete view of dinoflagellate responses to chemical stressors. Overcoming the complex genome of dinoflagellates and increasing their genomic resources is therefore essential to push further. The combination of OMICs studies will provide a much-needed global view of molecular processes, which is essential to optimize the production of dinoflagellate metabolites of interest and identify markers of HABs’ formation and toxin production events.

**Abstract:**

Dinoflagellates are important primary producers known to form Harmful Algae Blooms (HABs). In water, nutrient availability, pH, salinity and anthropogenic contamination constitute chemical stressors for them. The emergence of OMICs approaches propelled our understanding of dinoflagellates’ responses to stressors. However, in dinoflagellates, these approaches are still biased, as transcriptomic approaches are largely conducted compared to proteomic and metabolomic approaches. Furthermore, integrated OMICs approaches are just emerging. Here, we report recent contributions of the different OMICs approaches to the investigation of dinoflagellates’ responses to chemical stressors and discuss the current challenges we need to face to push studies further despite the lack of genomic resources available for dinoflagellates.

## 1. Introduction

Dinoflagellates are a diverse ecological group of phytoplankton. Among the 2000 dinoflagellate species, 90% are free-living in marine or fresh waters, 9% are parasitic and 1% are in symbiotic association with corals or invertebrates. Half of the species are heterotrophs, and the other half are autotrophs or mixotrophs feeding on both strategies [1,2]. Therefore, dinoflagellates are the second most prominent primary producer behind diatoms [3] but are also known to form Harmful Algae Blooms (HABs) or produce toxins. For example, the *Alexandrium* genus produces saxitoxin, which is responsible for Paralytic Shellfish Poisoning in humans. Thus, some dinoflagellates may have consequences on the economy, health and ecosystems [4].

Seawater chemical variations may induce chemical stressors that influence HABs’ formation and toxins’ production. Therefore, studying dinoflagellate responses to chemical stressors is crucial to understand and eventually prevent such events. Chemical stressors are elements or molecules that modify cell homeostasis through photosynthesis, energy metabolism or cell wall fluidity disruptions and that induce oxidative responses that could lead to cell death (Table 1). In aquatic ecosystems, chemical stressors faced by free-living dinoflagellates are nutrient availability, pH, salinity and anthropogenic pollution such as nanoplastics [5] or trace metals [6]. To study the responses of dinoflagellates under stressful conditions, photosynthesis, pigments, reactive oxygen species (ROS), toxins and growth parameters are monitored. For example, micro and nanoplastic exposure represses growth, inhibits photosynthesis and induces ROS and toxin production in *Amphidinium carterae* [5]. While ecophysiological approaches do not identify the molecular mechanisms underpinning physiological responses, the emergence of OMICs analyses offers powerful tools to explore the genome, transcriptome, proteome and metabolome responses to chemical stressors.

In comparison with other microalgae (e.g., diatoms, or green algae) [7,8,9,10], the dinoflagellate genome is larger and more complex owing to its high recombination rate and high proportion of repeats, mostly retrotransposons [11]. Their genome size is strikingly variable between species, ranking from 0.6 Gb in the Symbiodiniaceae *Symbiodinium fitti* [12] to 185 Gb in *Lingulodinium polyedrum* [11], while the human genome has 3 Gb [13] and current model microalgae, *Phaeodactylum tricornutum*, *Thalassiosira pseudonana* and *Chlamydomonas reinhardtii*, have 27.4 Mb, 32.4 Mb and 121 Mb, respectively [9,10]. Furthermore, the dinoflagellate genome exhibits unique spatial organization. Chromosomes are highly and permanently condensed in a cholesteric liquid crystalline structure and are lacking nucleosomes [11]. Some species can reach 94 chromosomes in total [14], and chromosomes are organized in more Topologically Associated Domains than other eukaryotes [15]. These features suggest unique genome regulation and make the dinoflagellate genome resolution low. Incomplete genome sequencing is detrimental to transcript and protein identification. Thus, genomic studies assessing dinoflagellate responses to chemical stressors remain scarce. For example, 19 protein spots remained unidentified in a proteomic study on nitrogen deficiency stress response using 2D electrophoresis in *Prorocentrum shikokuense* (*syn. P. dongaihense*) [16]. Therefore, the lack of transcriptomic and proteomic data restrains the understanding of the regulation of molecular responses to chemical stressors.

This review aims to analyze recent OMICs studies and evaluate their integrations and contributions to physiological studies, to highlight their limits regarding the complex genome of dinoflagellates and also to identify the remaining gaps to tackle dinoflagellate responses to chemical stressors.

## 2. Transcriptomic Approaches

The complexity of the dinoflagellate genome and especially the high amount of repeats makes the analysis of a whole genome sequence difficult. During the past decade, transcriptomics has become a central technology to assess the regulation of known or unknown genes [17]. High-throughput technologies have been developed to quantify transcripts’ expression levels. The low RNA sample requirement and increasingly accessible cost of these technologies make them a method of choice to study the response of non-models.

**Table 1:** Recent OMICs studies on dinoflagellate responses to chemical stressors.

Purpose	Species	Approach	Findings	Ref
**HAB formation**				
Effects of N-limitation to understand Harmful Algae Blooms’ (HABs’) formation	*Prorocentrum shikokuense*	RNA-seq	N-uptake, N-recycling and a shift to mixotrophy constitute a strategy to cope with N-deficiency	[18]
Responses of membrane proteome to metal contamination in a harmful species	*Alexandrium pacificum*	2D-electrophoresis	The downregulation of metal-binding transporter is a strategy to limit metal entry in the cell The ATP synthase downregulation is a strategy to reduce oxidative stress	[19]
Proteomic responses to phosphorus deficiency to understand HABs’ formation	*Alexandrium catenella*	iTRAQ-based quantitative proteomic	Carbon accumulation through starch polymerization, the utilization of Glucose-6-Phosphate as a dissolved organic phosphorus and carbon source, and a reduction in phosphorus demand in response to phosphorus deficiency	[20]
Transcriptional and post-transcriptional regulation under nutrient addition on a bloom-forming species	*Prorocentrum shikokuense (syn. P. dongaihense)*	RNA-seq and microRNA sequencing	N- and P-metabolism, energy and carbohydrate metabolisms, cell division and microbial defense are upregulated at the transcript level under blooming conditions, while cell wall remodeling, amino acid metabolism and reactive oxygen species production might be regulated by micro-RNA	[21]
Metabolomic changes in response to bacterial algicide IRI-160AA	*Karlodinium veneficum*	LC-MS/MS	Increase in oxidative stress biomarkers, antioxidants and compounds involved in DNA damage and the programmed pathway leading to cell death	[22]
**Impact of anthropic pressures**				
Proteomic responses to lead, zinc, copper and cadmium contamination	*Alexandrium pacificum*	2D-electrophoresis	Photosynthesis ability and oxidative stress response decrease energy metabolism, protein translation and degradation. In addition, proteolytic activity is downregulated under metal stress	[23]
**Symbiodiniaceae mechanisms**				
Effect of trace metal deficiencies	*Fugacium kawagutii*	RNA-seq	Trace metal might alter adhesion abilities and cause an immunity response Evidence for a tradeoff between iron demand and oxidative stress response	[24]
Nanoplastic effects on two *Symbiodiniaceae* species	*Symbiodinium tridacnidorum* *Cladocopium* sp.	RNA-seq	Nanoplastic affect photosynthesis efficiency and mitosis; it decreases intracellular degradation and increases motility The sensitivity to nanoplastic exposure is species-specific	[25]
Metabolomic changes to acidification	*Breviolum minutum*	LC-MS/MS	Acidification affects biosynthesis of amino acids and proteins Accumulation of saturated fatty acids and oligosaccharides is enhanced as a strategy to cope with acidification	[26]
Physiological and proteomic response to nutrient stress	*Symbiodinium* *microadriaticum*	LC–MS/MS	Proteomes were strongly affected by phosphate limitation. Very high N:P inhibited *Symbiodinium* cell division while increasing the abundance of chloroplast proteins	[27]
Comparison of nutrient availability effect in symbiont physiology in culture and in hospite	*Breviolum minutum*	LC-MS/MS	Photosystem proteins, antioxidant proteins and multicopper oxidase notably increased in abundance in the high-nutrient regimes, irrespective of the *B. minutum* state. In hospite vs. the free-living state, an increase in proteins involved in phosphoinositol metabolism potentially reflects inter-partner signaling that regulates the symbiosis	[28]
**Toxin production**				
Metabolomic changes in a toxic dinoflagellate to salinity stress	*Dinophysis sacculus*	LC-MS/MS and LC-HRMS/MS	Non-significant changes in pectenotoxin (PTX), okaidaic acid and osmolyte under different salinity concentrations suggest a high tolerance to salinity variation in *Dinophysis sacculus*	[29]
Identification of new toxin analogs	*Dinophysis strains*	HRMS and Molecular Networking	Metabolites’ patterns are species-specific Identification of 5 putative new toxins analogs	[30]
Genetic regulation of metabolites’ production under phosphorus and nitrogen starvation	*Amphidinium gibbosum*	Illumina Miseq, Iso-seq, RNA-seq, microRNA-seq	Involvement of post-transcriptional regulation through microRNA, alternative splicing and polycystronic expression of specific metabolites’ production	[31]
**Bioproduction optimization**				
Lipid profile under nutrient deficiency and algicidal bacterium to optimize biofuel production	*Prorocentrum shikokuense (syn. P. dongaihense)*	GC-MS	N and P stress induce lipid accumulation and change lipid properties to match the biodiesel standards	[32]
Lipid content, docosahexaenoic acid (DHA) productivity, fatty acid composition and metabolomic analysis under different nitrogen-feeding strategies	*Crypthecodinium cohnii*	GC-MS	Heterotrophic culture conditions may alleviate high-nitrogen inhibition effect to induce higher DHA productivity as well as changes in amino acids, polysaccharides, purines and pentose phosphate pathway	[33]
Metabolomic changes due to chemical modulators	*Crypthecodinium cohnii*	Targeted LC-MS	The chemical modulators: naphthoxyacetic acid, salicylic acid, abscisic acid and ethanolamine increased lipid accumulation. The enhanced metabolism in glycolysis and tricarboxylic acid cycle as well as the decreased metabolism in pentose phosphate pathway are related to the increased lipid biosynthesis	[34]

**Figure 1:**
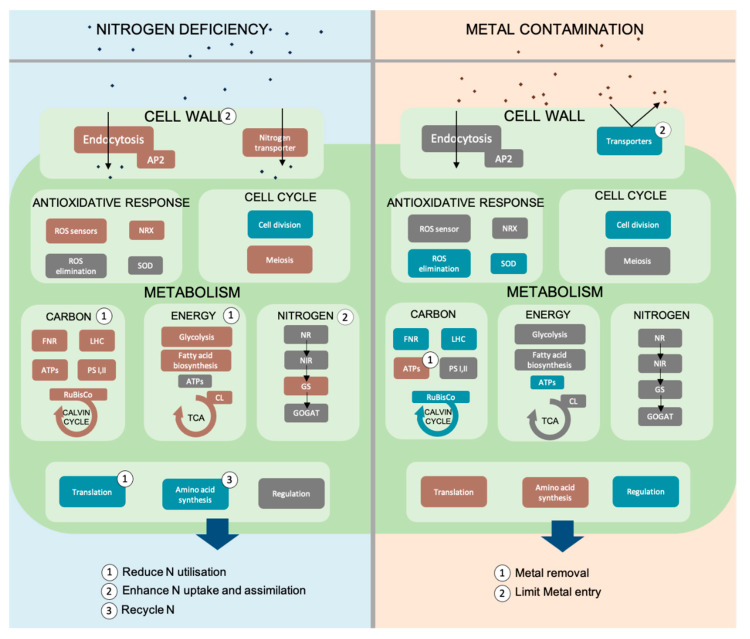
Schematic synthesis of metabolic pathways and strategies under nitrogen deficiency (**left**) or metal stress (**right**) in *Karenia mikimotoi*, *Prorocentrum shikokuense* and *Alexandrium pacificum* based on transcriptomic and proteomic analysis. Brown: enhanced metabolic pathways factor; gray: unchanged metabolic pathways; blue: inhibited metabolic pathways. Numbers refer to strategies. AP2: adaptor protein involved in clathrin-dependent endocytosis; NRX: nucleoredoxin involved in ROS-mediated signaling pathways; SOD: superoxide dismutase involved in antioxidant response to ROS; FNR: ferredoxin NADP(+) reductase involved in electron transfer in photosynthetic chain; LHC: Light-Harvesting Complex involved in chlorophyll collection and transfer to photosystems; ATPs: ATP synthase; PSI, II: photosystems I and II; TCA: tricarboxylic acid cycle; CL: citrate lyase involved in the tricarboxylic acid cycle; NR: nitrate reductase; NiR: nitrite reductase; GS: glutamine synthase; GOGAT: glutamate synthase; N: nitrogen.

Organisms such as dinoflagellates [35]. RNA-seq analyses are currently the most utilized technology to quantify the expression level of transcripts in dinoflagellates. The common approach is to compare differentially expressed genes’ and pathways’ enrichment analyses under control and stress conditions. This approach has been conducted under nutrient starvation conditions and with anthropogenic contaminants such as nanoplastics and trace metals across various dinoflagellate species including toxic, HAB-associated and free-living species (Table 1) Nutrient stress, such as nitrate and/or phosphorus deficiencies, impacts gene expression; they constitute major macronutrients that can limit dinoflagellates’ growth and are involved in several cellular functions such as amino acid metabolism or cell signaling [36]. The harmful dinoflagellate, *Karenia mikimotoi*, improves its N-assimilation capacity using multiple pathways and N sources, such as the nitrogen economy, and N reuse from proteins that constitute a comprehensive strategy to cope with N deficiency (Figure 1) [37]. Similarly, *Prorocentrum shikokuense* reuses nitrogen from urea and amino acid origins [18]. In addition, the upregulation of enzymes implied in C4 metabolism and the Calvin cycle suggests a carbon accumulation strategy under N deficiency (Figure 1). This mechanism might allow for rapid growth when the environment shifts to N-repleted conditions. To cope with phosphorus deficiency, it is well established that dinoflagellates switch to a dissolved organic phosphorus (DOP) source when the dissolved inorganic phosphorus (DIP) availability is low (Table 1). Recent studies show that the molecular mechanisms underlying this strategy are variable depending on species [18,38,39]. Phospholipid catabolism is enhanced in *Prorocentrum shikokuense (syn. P. dongaihense)* via the upregulation of phospholipid-transporting ATPase [38] (Table 1). Endocytosis is suggested as a mechanism to enhance nitrogen and phosphorus uptake and as a heterotrophic strategy in *Prorocentrum shikokuense* [18] and *Prorocentrum shikokuense (syn. P. dongaihense)* [39] (Figure 1). The increase in cell motility has also been suggested as a heterotrophic strategy to supplement phosphorus acquisition by facilitating predation [39]. Transcriptomic responses highlight trade-offs between cellular functions and energy reallocations to cope with nutrient deficiencies (Table 1).

While a reallocation of iron in the synthesis of iron-containing superoxide dismutase might be a trade-off strategy in the Symbiodiniacea, *Fugacium kawagutii* [24], to cope with ROS accumulation under iron deficiency, numerous transcriptional modifications under chemical stressors are still poorly understood. It is currently suggested that energy is reallocated toward sexual reproduction under an adverse environment rather than toward toxin production (Table 1). Meiosis is inhibited under phosphorus depletion in *Fugacium kawagutii* [40]. Furthermore, the roles of toxins in stress responses need to be further investigated to fully understand this trade-off. In addition, emerging harmful pollutants require further investigations, such as micro- and nanoplastics, which may cause oxidative damages and apoptosis to dinoflagellates [41]. The downregulation of cell surface receptors involved in cell dissociation suggests that nanoplastic exposure might cause the disruption of symbiosis in corals [25]. Despite the dozen of studies available, transcriptomic responses of dinoflagellates to micro and nanoparticles remain poorly investigated, and current studies are focusing on the physiological responses of dinoflagellates to these pollutants [42,43].

To summarize, cell wall composition or dynamic, energy metabolism, metabolic landscape, oxidative response, cell signaling, immunity and cell cycle are regulated in response to chemical stressors. Altogether, these cellular processes form a comprehensive strategy to cope with chemical stressors that ecophysiological studies cannot disentangle (Figure 1). However, even if the overall transcriptional rate of dinoflagellates in response to stress highlights the importance of post-transcriptional regulation in dinoflagellate responses [24], proteomic analysis, by characterizing the post-transcriptional and translational regulation, is required to provide a complete comprehension at the different genome scales of dinoflagellate responses to environmental stress.

## 3. Proteomic Approaches

Proteins are involved in cell structure and metabolism and can affect the cell response to the environment. Initially, dinoflagellate proteomic studies relied mostly on 2D electrophoresis protein separation [18], but overlapping proteins are not well identified, and only hundreds of proteins can be analyzed at a time. The emergence of high-throughput proteomics using mass spectrometry enables the identification of thousands of proteins and the development of protein databases for dinoflagellate proteins (Table 1). This facilitates the investigation of proteome modifications under stress conditions [19]. Recent studies notably focused on dinoflagellate responses to nutrient starvations. In *Prorocentrum shikokuense (syn. P. dongaihense)*, proteins involved in photosynthesis, carbon fixation amd protein and lipid synthesis are downregulated under nitrogen deficiency, while nitrogen reallocation and transport activity proteins are upregulated [16]. Consistently with the transcriptomic responses, the recycling strategy to cope with nitrogen shortages operates over proteins’ degradation in *P. shikokuense (syn. P. dongaihense)* (Figure 1) [16]. Under phosphorus deficiencies, the demand of P is reduced via the replacement of phospholipids by glycosphingolipids in *Alexandrium catenella*. The trend of shifting from DIP sources to DOP sources under phosphorus stress is also suggested by the proteome responses. Glucose-6-Phosphate might be used as an alternative to DOP and carbon sources when phosphorus is deficient in *A. catenella* [20]. In parallel, the carbon content maintenance strategy under nutrient deficiency, operating through starch polymerization in *A. catenella* [20], seems to be managed by an unknown mechanism in *P. shikokuense (syn. P. dongaihense)* [16]. Photosynthesis inhibition, suggested at the transcript level under N deficiency, is also observed at the protein level through photosynthesis-related protein downregulation, whereas under P deficiency, in *A. catenella*, the upregulation of photosystem-I- (PSI) and photosystem II (PSII)-associated proteins suggests a compensatory mechanism to the decrease in photosynthesis, consistently with the physiological maintenance of the photosynthetic efficiency [20]. The oxidative stress response contributes to defensive strategies in response to nutrient stress. In *P. shikokuense (syn. P. dongaihense)*, the upregulation of a redox-sensitive protein (NRX) under nitrogen-depleted conditions suggests the enhancement of ROS elimination as a defensive strategy (Figure 1) [16].

In response to metal stress, both soluble and membrane proteomes are modified in two *Alexandrium* species and suggest a decrease in photosynthesis efficiency [19,44] (Figure 1). In *A. catenella*, the ATP synthase upregulation is proposed as a compensatory effect of the overall proteome downregulation and photosynthesis reduction as a strategy to improve energy production and metal removal from the cell [44] (Figure 1). Conversely, in *A. pacificum*, the ATP synthase subunit is downregulated and the subsequent ATP synthesis reduction may constitute a strategy to limit oxidative stress. Additionally, the downregulation of the scramblase protein may be an adaptive response to decrease metal uptake [19] (Figure 1). Regarding defensive strategies under zinc and lead stress, oxidative stress proteins are downregulated (e.g., proteasome subunit) [44].

To summarize, the proteome responses to chemical stressors include cell wall modifications, metabolism landscape remodeling, oxidative response and energy metabolism adaptation and provide a comprehensive strategy to cope with these stressors (Figure 1). Therefore, these studies clarified some species-specific molecular mechanisms to cope with chemical stressors. However, proteomic studies under chemical stress conditions remain scarce compared to transcriptomic studies (Table 1). Some chemical factors studied at the transcriptomic level are underexplored at the proteomic level (e.g., micro- and nano-plastic studies). To fully understand dinoflagellate responses under stress conditions, metabolites are important molecules to consider, especially when their biosynthetic pathways are poorly identified.

## 4. Metabolomic Approaches

Metabolomics is emerging as an important OMICs tool to supplement genomic, transcriptomic and proteomic analyses of biological systems by qualitatively and/or quantitatively analyzing metabolite profiles. Metabolites are molecules with different structures and functions within the cell. They include sugars and lipids that are used in cell signaling and cell energy metabolism but also specific compounds such as phycotoxins produced by dinoflagellates under given conditions that are still poorly understood. Because metabolites’ identification does not rely directly on genome sequences but on the chemical structure of molecules, metabolomic approaches are more global and efficient on non-model organisms such as dinoflagellates. However, metabolomic studies on dinoflagellates are still in their early stages. Some recent targeted metabolomic studies analyze toxins’ production under nutrient and pH variations and lipid production under nitrogen or phosphorus availabilities or identify essential metabolites for coral–dinoflagellate symbiosis [32,45,46] (Table 1). Alongside this, untargeted metabolomics are promising approaches to identify biomarkers of HABs or pollutions [47,48,49].

Regarding the effect of chemical stressors, acidification may affect the biosynthesis of amino acids and proteins and thereby inhibit *Breviolum minutum* growth [28]. However, the accumulation of saturated fatty acid and oligosaccharides might be a defense strategy to minimize ROS damage on cell membranes [28]. Nitrogen-feeding strategies may influence the productivity of high-value products such as docosahexaenoic acid (DHA, e.g., *Crypthecodinium cohnii* under heterotrophic culture conditions; Table 1). Metabolomic analyses demonstrate large numbers of amino acids, polysaccharides and purines are upregulated in *C. cohnii* and highlight the mechanism of high nitrogen inhibition [28]. In the same species, chemical modulators, naphthoxyacetic acid (BNOA), salicylic acid (SA), abscisic acid (ABA) and ethanolamine (ETA), increased lipid accumulation. Targeted metabolomic approaches show that the enhanced metabolism in glycolysis and the tricarboxylic acid cycle, as well as the decreased metabolism in the pentose phosphate pathway, are important for the cumulative effects of BNOA and ETA and SA and ETA on lipid accumulation [33]. On the harmful and mixotrophic dinoflagellate, *Karlodinium veneficum*, a species that produces hemolytic, ichthyotoxic and cytotoxic karlotoxins, the metabolomics analysis of cells exposed to the algicidal bacterium (IRI-160AA) shows the upregulation of oxidative stress biomarkers, antioxidants and metabolites known to induce DNA damage and pathways leading to cell death [22]. With another well-known harmful algae genus, *Dinophysis*, the response of their metabolome to salinity stress shows strain-dependent modifications under different salinity concentrations. These results, as well as the absence of effects on growth rate and toxins (okadaic acid and pectenotoxin), suggest that *Dinophysis sacculus* is resistant to salinity variations [29]. However, more studies are needed to explore dinoflagellate metabolomic responses to nutrient deficiency and metal contamination, as already implemented with transcriptomic and proteomic approaches.

Recently, metabolomic molecular networking using an untargeted approach that clusters chemical species with similar MS/MS spectrums has been used to identify new toxins in *Dinophysis* species [30]. Applying this method under different chemical stress conditions would improve our knowledge of the set of metabolites known to be involved in the stress response of dinoflagellates. Despite metabolite databases [50], the specific metabolite diversity of dinoflagellates is still poorly recorded. Therefore, the development of metabolomic methods aims to increase the efficiency of identifying specific metabolites [51]. For example, the use of updated computational tools to integrate untargeted and targeted metabolomics approaches achieved an increase of 40% in the amount of lipid annotation from a previous study on microalgae [52]. The increasing use of metabolomics is promising to enrich our understanding of the involvement of specific dinoflagellate metabolites in their response to chemical stressors. The availability of the different OMICs datasets offers crucial information for analyses. However, these approaches are conducted separately, making the confrontation and integration of results tedious and time-consuming. Therefore, integrating OMICs datasets is a challenge to face in order to visualize the multi-scale genomic response of dinoflagellates to different environmental drivers.

## 5. Multi-OMIC Approaches

Integrated OMICs studies use different OMICs approaches in a single study. They are interesting to improve our understanding of the different responses of an organism to chemical stressors and to confirm or over-ride inferences made at each OMIC response level. Only a few studies on dinoflagellates integrate OMICs datasets. One approach is to investigate post-transcriptional regulation in response to nutrient deficiencies through the integration of transcriptome and microRNAome datasets. For example, in a study on the HAB-associated species, *Amphidinium gibbosum* (Table 1), no toxin biosynthesis-related transcripts were differentially expressed in the transcriptome in response to nitrogen and phosphorus starvations. However, miRNA-targeting precursors of toxins were upregulated during nitrogen starvation, suggesting a miRNA-mediated post-transcriptional regulation of toxins’ biosynthesis in response to nutrient deficiencies [31]. The integration of miRNA datasets to transcriptomes highlights the importance of the post-transcriptional regulation of dinoflagellate responses to chemical stressors and avoids false assumptions made on transcriptome analyses only. Importantly, tools are developed to integrate OMICs datasets and enable us to identify biomarkers among the different layers of a biological system (e.g., MOTA [53]). Applying such method to dinoflagellate OMICs datasets obtained under chemical stress conditions could help identify HABs related to pollution events. The genome complexity of dinoflagellates makes genomic resources scarce [54]. These obstacles explain the bias toward transcriptomic analysis among the OMIC approaches. Improving the overall understanding of the stress response of dinoflagellates requires improvements in the quality of genomic resources. With the emergence of NGS, genomic resources across dinoflagellates species has increased. Hence, among the 20 Symbiodiniaceae genomes, 18 have been published since 2018 [55], and the de novo assembly of transcriptomes has multiplied. Gene sets of sequenced genomes are improved through new genomes’ assembly. In *Fugacium kawagutii*, 11,984 new protein-coding genes were identified in a new genome assembly [24]. To centralize these resources, the Symbiodiniaceae and Algal Genomic Resources database (SAGER) has been created [56]. In parallel, bottom-up approaches using mass spectrometry to validate protein identifications emerged as methods to improve transcriptome and ultimately genome resolutions [57].

OMIC studies unveil a diversity of molecular mechanisms used to cope with chemical stressors. However, most of the mechanisms remain untested. This is mainly due to the lack of genetic tools in dinoflagellates. Nuclear and plastid transformation methods are starting to be implemented on dinoflagellates and are promising tools to conduct functional genomic studies on these species [58,59]. More recently, gene knockdown methods using morpholinos have been designed for dinoflagellates [60]. Integrating big datasets from single OMICs analyses remains challenging. Yet, these advances should help to explain genetic regulation responses to chemical stressors. The low correlation between transcriptomic and proteomic results on dinoflagellates suggests that post-transcriptional regulation is a key to study molecular responses and calls for more proteomic and miRNA studies. Also, the overall low transcriptomic responses recorded in chemical stress conditions [24,37,61] mean we cannot exclude the role of non-coding sequences. The impact of chemical stressors on genomic responses of dinoflagellates will remain unclear until the role of ‘junk’ DNA and large amounts of repeats is elucidated.

## 6. Conclusions

Multi-OMICs approaches coupled with ecophysiological studies could thus play a major role in improving our knowledge of the mechanisms related to chemical stresses in intricate marine organisms. This is especially relevant to dinoflagellates, which can lead to HABs with great impacts on health, economies and ecosystems.

## Data Availability

Not applicable.
